# The Parkinson's Disease Progression Neuroimaging Initiative

**DOI:** 10.1155/2021/2230196

**Published:** 2021-12-30

**Authors:** Shiyi Zhu, Zizhao Ju, Ping Wu, Fengtao Liu, Jingjie Ge, Huiwei Zhang, Jiaying Lu, Ling Li, Min Wang, Jiehui Jiang, Jian Wang, Chuantao Zuo

**Affiliations:** ^1^School of Communication and Information Engineering, Shanghai University, Shanghai, China; ^2^PET Center and National Research Center for Aging and Medicine & National Center for Neurological Disorders, Huashan Hospital, Fudan University, Shanghai, China; ^3^Department of Neurology and National Research Center for Aging and Medicine & National Center for Neurological Disorders, Huashan Hospital, Fudan University, Shanghai, China; ^4^School of Life Science, Shanghai University, Shanghai, China

## Abstract

The Parkinson's Disease Progressive Neuroimaging Initiative (PDPNI) is a longitudinal observational clinical study. In PDPNI, the clinical and imaging data of patients diagnosed with Parkinsonian syndromes and Idiopathic rapid eye movement sleep behavior disorder (RBD) were longitudinally followed every two years, aiming to identify progression biomarkers of Parkinsonian syndromes through functional imaging modalities including FDG-PET, DAT-PET imaging, ASL MRI, and fMRI, as well as the treatment conditions, clinical symptoms, and clinical assessment results of patients. From February 2012 to March 2019, 224 subjects (including 48 healthy subjects and 176 patients with confirmed PDS) have been enrolled in PDPNI. The detailed clinical information and clinical assessment scores of all subjects were collected by neurologists from Huashan Hospital, Fudan University. All subjects enrolled in PDPNI were scanned with ^18^F-FDG PET, ^11^C-CFT PET, and MRI scan sequence. All data were collected in strict accordance with standardized data collection protocols.

## 1. Introduction

Parkinsonian syndromes are a group of diseases featured by symptoms of parkinsonism, including bradykinesia, rigidity, tremor, and postural instability [1]. Parkinsonian syndromes include at least dozens of diseases, including MSA, PSP, and CBD. Parkinson's disease (PD) is the most common neurodegenerative parkinsonian syndrome, affecting approximately 2% to 3% of adults over the age of 65 [2]. The main pathological manifestations of PD are the degeneration of dopaminergic neurons in the substantia nigra, the reduction of striatal dopamine, and the abnormal aggregation of *α*-synuclein. Aside from PD, there are a number of other diseases, which also present with Parkinsonian features, but its further clinical manifestations are atypical for PD. It has been named as atypical parkinsonian syndromes (APS) or atypical parkinsonism. APS mainly include multisystem atrophy (MSA), progressive supranuclear palsy (PSP), corticobasal degeneration (CBD), and dementia with Lewy bodies (DLB). The pathogenesis, clinical manifestations, and treatment methods of PD and APS are different. APS are synucleinopathies and tauopathies, and its clinical characteristics depend on the site of abnormal protein *α*-synuclein and tau deposition [3]. Several studies have demonstrated that RBD, a rapid eye movement sleep- (REM-) phase-associated parasomnia with the loss of muscle atonia and the occurrence of abnormal movements during REM [4], is a prodromal phase of synucleinopathies disease including PD, DLB, and MSA, which has up to 30% estimated risk of synucleinopathy at 3 years of disease duration, increasing to 76% at 10 years and coming to about 91% at 14 years [5, 6]. RBD patients can also present with clinical features of synucleinopathies, such as deficits in motor, cognitive, olfaction, color discrimination, and autonomic functions [7]. Up to now, the prediction of the risk and time course of individual subsequent phenoconversion is still challenging, thus an increasing number of neuroimaging researches have been carried out, for the purpose of improving the understanding of the development and progression of RBD and related neurodegenerative diseases.

The diagnosis of Parkinsonian syndromes relies mostly on clinical criteria. However, in lack of specific clinical signs initially, it can be difficult in the differential diagnosis among these diseases especially at an early phase. Moreover, the clinical symptoms could develop heterogeneously during the progression of these diseases. Therefore, it is necessary to establish a clinical database for parkinsonian syndromes, which can manage and analyze medical records for Parkinsonian syndromes patients, including clinical, imaging, and biospecimen data and clinical scale evaluation; individualized therapies enhance the diagnosis and treatment trials on PDS, find the novel imaging manifestations and clinical biomarkers of PDS, promote the accuracy of the diagnosis and differentiation, and enhance the development of PD treatment. There are several databases of PD and Parkinsonian syndrome have been established worldwide, and the most widely used one is the international multicenter study called PPMI (the Parkinson's progression markers initiative), which was a multicenter study enrolled in 24 sites, which started in June 2010. The cohort included 423 untreated PD patients, 196 healthy control (HC) subjects, and 64 SWEDD (scans without evidence of dopaminergic deficit) subjects. The study established standardized protocols for the acquisition of clinical and neuroimaging data, which can provide basis for further PD research [8]. And PPMI's clinical research data and standardized protocols can be obtained on their official website (http://www.ppmi-info.org). Parkinson's Disease and Movement Disorders Multicenter Database and Collaboration Network in China (PD-MDCNC) (http://pd-mdcnc.com/zh-cn/normal.html) is a database comprised of several cohorts of PD and other movement disorders, established by Xiangya Hospital Central South University in 2018, and has already enrolled over 100000 cases from 100 sites until 2020, including the Chinese Parkinson's disease registry (CPDR), the Chinese familial Parkinson's disease registry (CFPDR), the early onset Parkinson's disease registry (CEOPDR), the Chinese Parkinson's disease with GBA Variants registry (PD-GBAR), the Chinese Parkinson's disease with LRRK2 Variants (CPD-LRRK2R), the Chinese Parkinson's disease with parkin Variants registry (CPD-PARKINR), the Chinese essential tremor registry (CETR), Dystonia, PSP, MSA, CBD, and health control. The data contains demographic characteristics, clinical symptoms, environmental factors, family history, comorbid phenomena, brain imaging, genomics, treatment options, neuropsychology, and quality of life assessment. PD-MDCNC mainly focuses on the clinical features, comorbidities, progression and prognosis of PD, and other movement disorders among Chinese people. But the summarized data of the PSP/MSA/CBD cohort is still unavailable on the website. These cohorts are helpful to the diagnosis and differentiation of PD at early phase.

In the past decades, researchers conducted dopaminergic or metabolic PET imaging to assist in the diagnosis of PD in several studies, and ^11^C-CFT and ^18^F-FDG PET scans have been proved to be objective tools for the evaluation of disease severity and treatment efficacy [9, 10]. Meanwhile, previous studies have illustrated the correlations between functional imaging measures and clinical ratings [11, 12]. Thus, with the combination of dopaminergic, metabolic imaging, MRI, and clinical ratings and examining the correlations between them, the interaction among neuroimaging and clinical manifestations may be further illuminated, and it may provide credible biomarkers for the diagnosis and curative effect evaluation.

Here, the details of the design of PDPNI were summarized. This is an ongoing longitudinal observational study launched by PET Center and Department of Neurology, Huashan Hospital, Fudan University. The main aim of PDPNI is to identify PDS progression biomarkers, help the diagnosis result more accurate in patients with uncertain Parkinsonism, and provide new clues for the treatment of Parkinsonism. The study included the PD\RBD\PSP\MSA cohort for long-term longitudinal follow-up. Patients enrolled in this study all went through functional imaging modalities including FDG-PET, DAT-PET imaging, ASL MRI, and fMRI, as well as cognitive assessment and motor function assessment. All of the data was sorted and uploaded to the database. The database was able to classify and sort patient data and download the corresponding category to meet needs.

## 2. Methods

### 2.1. Study Organization and Governance

**Figure 1:**
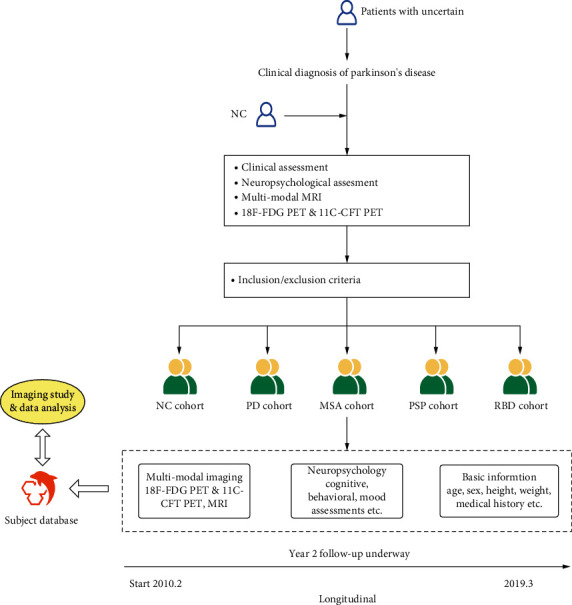
Overview of PDPNI study process.

PDPNI was launched by PET Center and Department of Neurology, Huashan Hospital, Fudan University. The neuroradiologists of PET Center were responsible for scanning, imaging interpretation, and reporting; the neurologists of Huashan Hospital were responsible for the enrolment, diagnosis, and clinical assessment of subjects; and students from the School of Communication and Information Engineering of Shanghai University were in charge of the construction and maintenance of website. The database construction were shown in Figure 1. The enrolment of all subjects was based on the China-US Biomedical Collaborative Research Program (No. 81361120393).

PDPNI was a prospective cohort comprised of healthy middle-aged and elderly patients and Parkinson's syndrome patients. The figure above is an overview of the PDPNI study, each of which has subdivision test items (Table 1) to indicate cognitive assessment, pathology detailed inspection items of information, and image data. After ten years of longitudinal tracking, four cohorts were obtained.

### 2.2. PDPNI Study Design

Subjects have been enrolled in this research since February 2010. PDS subjects will be recruited at disease threshold. All the patients were diagnosed by a movement disorder specialist in Huashan Hospital affiliated to Fudan University based on the clinical diagnostic criteria [13–17]. Those subjects screened as potential PDS subjects who were ineligible due to DAT scan without evidence of dopaminergic deficit (SWEDD) were eligible to be enrolled in a SWEDD cohort. However, no one was enrolled in this cohort so far. All of them were scanned with 18F-fluorodeoxyglucose positron emission tomography (^18^F-FDG PET) for glucose metabolism assessment and ^11^C-2-*β*-carbomethoxy-3-*β*-(4-fluorophenyl) tropane positron emission tomography (^11^C-CFT PET) for presynaptic dopaminergic binding assessment, and part of them (these subjects all voluntarily undergo MRI examinations) were scanned with multimodal MRI including structural MRI (sMRI), arterial spin labelling perfusion (ASL), and functional MRI (fMRI). The detailed clinical scores of cognitive assessment and motor function assessment evaluated with clinical scales (Unified Parkinson's Disease Rating Scale (UPDRS), Montreal Cognitive Assessment (MoCA), Self-Rating Anxiety Scale (SAS), Self-Rating Depression Scale (SDS), and Progressive Supranuclear Palsy Rating Scale (PSPRS); Table 1) were collected in all patients. All subjects were longitudinally followed every two years. Subjects underwent clinical assessment and imaging modalities according to established standardized protocols. During the period of follow-up, if subjects in the health control group developed Parkinsonism symptoms, they would be excluded from the cohort. If the RBD patients developed PD or MSA, they would be enrolled into the corresponding cohort. If the RBD patients developed DLB, the follow-up would be terminated. The PD database was established in November 2020, by the School of Communication and Information Engineering, Shanghai University, and both clinical and imaging data would be uploaded into the specific database and are open to registered users. The enrollment is still underway; meanwhile, the follow-up of enrolled subjects will continue and the data will be available through the PDPNI web.

### 2.3. Inclusion/Exclusion Criteria

Patients participating in the PDPNI project all met the following criteria: (1) patients with uncertain clinical diagnosis of parkinsonism; (2) ≥45 years old; (3) Hoehn and Yahr ≤ 1; and (4) time interval between imaging and the final clinical diagnosis ≤ 2 years. HC subjects were excluded if they had chief complaint and objective evidence on cardiovascular, respiratory, hematological, and neurological diseases or participated in radiopharmaceutical clinical trial research in the past 2 years. All subjects were excluded with following characteristics: (1) treated with antipsychotics, atypical antipsychotics, antiemetics, or other anti-PD drugs (tetrabenzoxazine, diarrhea, etc.) within 12 months; (2) women might not be pregnant; (3) with history of stroke, brain trauma, or other possible factors which may result in structural brain injury; (4) with the presence of pacemakers, aneurysm clips, artificial heart valves, ear implants, metal fragments, or foreign objects in the eyes, skin, or body or any other known contraindication; (5) and characteristics of atypical Parkinson's syndrome (MSA, PSP, etc.), such as excessive autonomic symptoms, and abnormal eye movements.

### 2.4. Cognitive Assessment

The project mainly focused on the clinical and neuroimaging signs of PDS patients. Clinical evaluation was performed every 2 years by 2 senior specialists of movement disorders from Huashan hospital. Clinical assessments contained motor function assessment and cognitive assessment, including Movement Disorders Society-Unified Parkinson Disease Rating Scale (MDS-UPDRS) and Hoehn and Yahr scales, the Montreal Cognitive Assessment (MoCA), Rey-O copy test, Similarity test, Clock test, Stroop Color Word Test, Wired Test, Sign to digit conversion test, Rey-O delayed imitation test, Auditory word learning test, Boston Naming Test, Fluency test, and Semantic similarity test. Besides, Epworth Sleepiness Scale and a REM sleep behavior disorder (RBD) questionnaire for sleep behavior assessing were also included. All of the clinical and imaging data were integrated into the PDPNI database by professionals after training. PDPNI will serve as a sustainable source of information management of standardization research, research progress, and results.

### 2.5. PET Imaging

1. The scanning equipment used is Siemens Biograph 64 HD PET/CT (Siemens, Erlangen, Germany)
2. In preparation before scanning, all subjects should fast over 6 hours and withdrew anti-Parkinsonian and antipsychotics drugs for more than 12 hours before clinical assessment and each imaging acquisition. And other drugs that may affect glucose metabolism also need to withdrew over 12 h before the ^18^F-FDG PET imaging
3. The acquisition protocols of PET imaging are shown in Table 2.

Acquisition for patients was performed in a resting state in a quiet and dimly lit room to avoid sensory, auditory, and motor stimulation to the patient.

### 2.6. Magnetic Resonance Imaging (MRI)

1. The scanning equipment used is a 3-tesla GE Discovery MR750 Scanner (Milwaukee, WI, USA)
2. Precautions before scanning as follows: ensure that no metal objects are implanted or left in/on the patient's body. Ask the participants if they suffered from claustrophobia since claustrophobic may be anxious or even prone to have panic attacks during the scanning. Inform the participants that loud noises would occur during the scanning and not to move their body during the long-time examination
3. The acquisition protocols of MRI are shown in Table 3.

### 2.7. Statistical Analysis

All analyses were performed using SPSS 20 (SPSS Inc., Chicago, IL), and a 2-tailed *P* value of less than 0.05 was considered significant.

### 2.8. Data Storage

**Figure 2:**
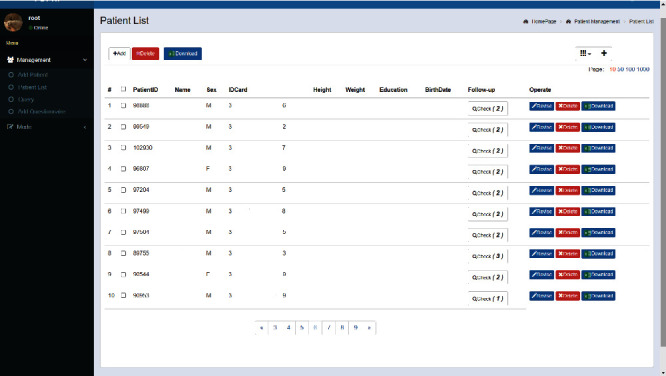
PDPNI website interface-patient information list.

A very important part of the PDPNI research is to achieve a cloud storage of PDS patient data and rapid data sharing. The PDPNI website is an essential component of the study as shown in Figure 2 (after hiding the patient's private information). The website will provide PDPNI data, including clinical and imaging data obtained during the long-term follow-up.

This website can help administrators realize the functions of uploading and downloading data of subjects and can make accurate queries based on characteristics and manage patient information more conveniently. Users requesting to query or download data need to register an account; fill in the name, gender, unit, email address, registration password, and mobile phone number to complete the registration; and then jump to the login page and enter the account password to login. Continuously update the data of the subjects in the cloud by connecting to the database, while uploading the image files for follow-up and directly downloading them remotely when needed, realizing intelligent management, which is safer and more efficient.

## 3. Results

The Parkinson's Disease Progression Neuroimaging Initiative (PDPNI) was launched in February 2010, enrolling 176 PDS patients (both inpatients and outpatients in Huashan Hospital affiliated to Fudan University). The tables below illustrate the sample distribution of the four cohorts and the characteristics of subjects (Tables 4 and 5). 48 healthy control subjects and 149 patients were followed up every two years, including 71/83 PD patients, 19/19 PSP patients, 23/23 MSA patients, and 36/51 RBD patients. A total of 295 cases of follow-up have been completed, including 134 cases of PD patients, 23 PSP, 31 MSA, 107 RBD, and 43 health control. All the patients were diagnosed by a movement disorder specialist in Huashan Hospital affiliated to Fudan University based on the clinical diagnostic criteria.

## 4. Discussion

The Parkinson's Disease Progression Neuroimaging Initiative (PDPNI) was launched in February 2010, by PET Centre Huashan Hospital affiliated to Fudan University, aiming at identifying PDS progression biomarkers through innovative techniques such as DAT PET imaging and MRI, therapeutic conditions, and clinical symptoms, hoping to promote the accuracy of diagnose in patients with uncertain Parkinsonism especially at early stage and promote the development of PD disease-modifying treatment trials and inform PD treatment. Four cohorts were established in this study, including PD, MSA, PSP, RBD, and health control (HC). The study has enrolled 224 subjects (176 PDS patients and 48 healthy subjects) from February 2010 to March 2019, and a total of 338 cases of follow-up were conducted. The follow-up is still ongoing, and more subjects will be enrolled in this project. Through statistical analysis of the age and gender of subjects in the four cohorts and healthy subjects, there are significant statistical differences between the HC cohort and the cohorts of PD/RBD/MSA. The follow-up of these four cohorts is still ongoing; we will supplement the data of these three cohorts in order to make the age and gender of each group of patients matched. All subjects went through the 18F-FDG PET and 11C-CFT PET scanning, and part of them were scanned with multimodal MRI. The detailed clinical scores of cognitive assessment and motor function assessment were collected and evaluated by neurologists of Huashan Hospital affiliated to Fudan University with clinical scales (UPDRS, MoCA, etc.). All of the study data was integrated into the database and collected strictly in accordance with standardized data collection protocols. Data was tracked vertically in strict accordance with standardized data collection protocols.

PDPNI may be of benefit to the research related to Parkinsonian syndromes. Several studies have been carried out based on PDPNI; the findings of these studies may provide novel perspectives in understanding the specific manifestations in PDS, provide new insights in the correlation between brain dysfunction and clinical features, and enhance the understanding of the pathological mechanism of PDS [18–23]. Thus, we believe these clinical evaluation data and image information of different types of patients can help develop more studies in the domain of Parkinsonian syndromes. It has already been demonstrated that regional changes in brain metabolism vary among parkinsonism and the differentiation among APS can be carried out by 18F-FDG-PET [24–28]. MRI technology can clearly show the anatomical structure of the substantia nigra and can reflect the pathological changes of PD through quantitative analysis [29]. Therefore, PDPNI data may be helpful in the studies of differential diagnosis. With the collection of treatment condition of patients with Parkinsonian syndromes, PDPNI may also be conducive to the update of the treatment plan for PD. Besides, the evaluation of cognitive assessment scales can help medical researchers evaluate the progression of the disease and further develop better treatment options. Except improving understanding of the pathophysiology and treatment of PD, PDPNI can also establish a standardized protocol for the acquisition, transmission, and analysis of clinical and image data for PD research. Continuous longitudinal follow-up of PD from the preclinical stage to the prodromal symptom stage, and finally to the stage of cognitive impairment, will provide a more profound understanding of the disease progression.

The PDPNI study is aimed at identifying PDS progression biomarkers through innovative techniques such as DAT PET imaging and MRI, therapeutic conditions and clinical symptoms, promoting the development of PD disease-modifying treatment trials, and providing new clues for PD treatment. At the same time, other Parkinson's syndromes such as PSP, MSA, and RBD, which are different from PD, are diagnosed on the basis of the clinical evaluation and images collected by PET and MRI. The follow-up of the clinical assessment and imaging of PDS patients and HC have been carried out, and all data was uploaded to the PDPNI database. It is a longitudinal study which has been running for over ten years, and the enrollment is still underway. A large amount of data could generate during long-term clinical monitoring of PD patients, which is not convenient for data management and statistical analysis. However, with the use of database, the collected data can be organized, processed, and sorted in a desired way. Integrating the basic clinical information, image data, and pathological information of different disease stages of patients into the database, doctors can obtain targeted clinical data of all patients. The database included the assessment scale data obtained from long-term follow-up and the DICOM images of PET and MRI, and all of the data can be downloaded and viewed directly and conveniently when needed. Besides, the follow-up of enrolled subjects will continue and the data will be available through the PDPNI website. The database can enhance the efficiency of the management and analysis of clinical imaging, clinical scale evaluation, individualized therapies, and other data for PD patients and make it convenient to observe the pathophysiological changes longitudinally. Therefore, PDPNI is a collaborative effort of specialists in the department of neurology, radiology, and nuclear medicine and IT technicians. In this paper, we comprehensively summarized the study methods, as well as the baseline data and follow-up data of the cohort obtained by advanced imaging techniques and professional clinical evaluation.

Different from other studies, the PDPNI study enrolled MSA, PSP, and RBD patients beside the PD cohort and conducted longitudinal cohort studies on them. In order to conduct a longitudinal study, all subjects of the project will be followed up for several years in PET center, Huashan Hospital. The same group of clinical assessment and functional imaging modalities were conducted every two years. Since PDPNI is a single center study, the sample size of PDPNI is small compared to the PPMI and PD-MDCNC study. But the follow-up is still ongoing, and more subjects will be enrolled in this project. We will update the data to form a more systematic PDS database. Another limitation is the sex ratio of the subjects and the age significance between cohorts. In the study, there were 224 subjects including healthy subjects, of which 150 were males and 74 were females. Apparently, there were more male volunteer researchers than females. In the follow-up study, we will consider the age range between different cohorts and the balance of the proportion of male and female volunteers when recruiting subjects. Thirdly, it is clear that biomarkers with a focus ranging from clinical symptoms to PD pathobiology to molecular genetic mechanisms are necessary for fully mapping PD progression. However, serum/CSF biomarkers such as *α*-synuclein, tau, and pathological biomarkers were not included in PDPNI so far. In the next phase of the study, we plan to collect the biospecimens including blood, CSF for plasma and CSF *α*-synuclein, and tau and conduct tau PET imaging in new subjects enrolled in this study after ethical approval, to provide useful and novel insights for PDS progression and diagnosis [30].

In summary, PDPNI is a cohort-rich longitudinal study. The subjects are healthy middle-aged people and people with Parkinson's syndrome. For the included subjects, follow-up data is collected every 1-2 years, and it is committed to advance the research progress of PD disease through the systematic analysis of a large amount of data. This process can also help each medical team complete the latest academic research and provide a large amount of clinical data.

The samples included in the clinical study are still being updated. In order to achieve better patient selection and stratification and to advance the research progress of Parkinson's syndrome, it is necessary to expand the cohort and further follow-up. In order to achieve the above goals, we will continue the follow-up data collection work and expand the sample size of baseline data and follow-up data.

## Figures and Tables

**Table 1:** Clinical examination items of PDPNI.

	Clinical assessments	Clinical information	Imaging data
Data collection	MoCA total score	Sex, age	FDG PET
	Rey-O copy test	Symptoms	CFT PET
	Similarity test	Medication compliance	ASL
	Clock test	MDS-UPDRS III	fMRI
	Stroop Color Word Test	HY classification	Structural MRI
	Wired Test	MSA	
	Sign to digit conversion test	PSPRS	
	Rey-O delayed imitation test	NMSS	
	Auditory word learning test	RBD self-evaluation	
	Boston Naming Test	ESS self-evaluation	
	Fluency test	PDQ39	
	Semantic similarity test	SAS	
		SDS	

**Table 2:** Acquisition protocols of PET imaging.

	11C-CFT PET imaging	18F-FDG PET imaging
Inject dose	350–400 MBq	150–200 MBq
Time duration between injection and scan	60-80 minutes	45-55 minutes
Scan mode	3D mode	3D mode
Reconstruction	OSEM method	OSEM method
Attenuation correction	CTAC	CTAC

OSEM: ordered subset expectation maximization; CTAC: computed tomography attenuation correction.

**Table 3:** Acquisition protocols of MRI.

	FOV	Slice thickness	Repetition time (TR)	Echo time (TE)	Inversion time (TI)	Flip angle
T1 Bravo	25.6 cm	1 mm	8.2 ms	3.2 ms	450 ms	12°
T2 Flair	24 cm	6 mm	2000 ms	28.4 ms	2100 ms	111°
fMRI	24 cm	3 mm	8800 ms	145 ms	/	77°
ASL	24 cm	4 mm	4844 ms	10.5 ms	/	/

**Table 4:** Sample distribution of research cohorts and healthy subjects.

Cohort	Number	Subjects were followed up	Cases of follow-up	Cases of only one follow-up	Cases of over twice follow-up
PSP	19	19	23	15	4
MSA	23	23	31	16	7
PD	83	71	134	32	39
RBD	51	36	107	5	31
HC	48	23	43	9	14

**Table 5:** Characteristics of subjects.

	HC (*n* = 48)	PD (*n* = 83)	PSP (*n* = 19)	MSA (*n* = 23)	RBD (*n* = 51)
Male (%)	24 (50.0)	53 (63.9)	16 (84.2)	15 (65.2)	40 (78.4)
Female (%)	24 (50.0)	30 (36.1)	3 (15.8)	8 (34.8)	11 (21.6)
Age (year ± SD)	61.9 ± 7.2	58.8 ± 9.5	64.7 ± 7.6	57.6 ± 8.0	66.1 ± 6.7
*P* value	NS	0.034∗	0.211	0.036^∗^	0.011^∗^

^∗^ *P* < 0.05. NS: not significant. Data are given as the mean ± standard deviation (SD) values.

## Data Availability

Due to restrictions on privacy, confidentiality, etc., all the pictures of the subject's information in the article have been encrypted. Due to various constraints, a “data sharing agreement” is required before the data is released. In terms of data sharing of PDPNI research, further review and approval may be required according to the specific research project request, and the data will be shared on the basis of the reviewed project.
